# Microscopic observation of dye molecules for solar cells on a titania surface

**DOI:** 10.1038/srep24616

**Published:** 2016-04-18

**Authors:** Shogo Koshiya, Shunsuke Yamashita, Koji Kimoto

**Affiliations:** 1 Surface Physics and Structure Unit, National Institute for Materials Science, 1-1 Namiki, Tsukuba, Ibaraki 305-0044, Japan; 2 Department of Applied Chemistry, Kyushu University, 1-1 Namiki, Tsukuba, Ibaraki 305-0044, Japan

## Abstract

The lateral distribution and coverage of Ru-based dye molecules, which are used for dye-sensitized solar cells (DSCs), were directly examined on a titania surface using high-resolution scanning transmission electron microscopy (STEM). The clean surface of a free-standing titania nanosheet was first confirmed with atomic resolution, and then, the nanosheet was used as a substrate. A single dye molecule on the titania nanosheet was visualized for the first time. The quantitative STEM images revealed an inhomogeneous dye-molecule distribution at the early stage of its absorption, i.e., the aggregation of the dye molecules. The majority of the titania surface was not covered by dye molecules, suggesting that optimization of the dye molecule distribution could yield further improvement of the DSC conversion efficiencies.

Dye-sensitized solar cells (DSCs) have attracted much attention because of their low production costs and easy fabrication compared with conventional solar cells^1^,^2^. Typical DSCs comprise a dye-coated semiconducting metal oxide photoelectrode, redox couple, and a counter electrode. The energy conversion efficiency of DSCs has been improved by developing new dye molecules and photoelectrodes as well as photoelectrode morphologies^2^,^3^,^4^. An energy conversion efficiency of higher than 10% was first demonstrated in 1993 by Grätzel *et al.* using Ru-based dye molecules and a titania photoelectrode^5^, and the highest conversion efficiency achieved thus far is 13%^6^. Further improvement of the conversion efficiency could be achieved through an investigation of the interface between the dye molecules and photoelectrode because the binding at the interface plays a key role in solar cell operation. Although the binding configurations between the photoelectrode surface and dye molecules have been studied using spectroscopic techniques^7^,^8^,^9^, few reports^10^,^11^ have examined the binding configurations or dye molecules on photoelectrodes. Transmission electron microscopy (TEM) was previously applied to examine the photoelectrode morphology^12^,^13^,^14^; however, the high-resolution observation of binding molecules was not achieved because of various experimental difficulties encountered in the specimen preparation and TEM observations. Thus, the distribution of dye molecules on a photoelectrode has not yet been fully investigated.

To realize direct observation of dye molecules on photoelectrodes with high resolution, first, a low-damage high-sensitivity electron microscopy approach must be developed. In this study, we utilized annular dark-field (ADF) imaging of scanning transmission electron microscopy (STEM), which is an effective technique to observe atomic arrangements with high sensitivity^15^,^16^,^17^. An aberration-corrected STEM instrument and liquid-nitrogen cooling specimen stage were used at an acceleration voltage of 80 kV to reduce specimen damage. We recently established a quantitative ADF imaging technique that enables the number of graphene layers to be counted using the quantitative ADF contrast^18^,^19^. In this study we adopt this highly sensitive and quantitative ADF imaging to dye-molecule observation.

STEM specimen preparation is the other challenge that must be overcome to realize the microscopic observation of dye molecules. Here, we use a titania nanosheet Ti_0.87_O_2_ as a photoelectrode, which is a two-dimensional crystal prepared by soft chemical delamination of a layered titanate crystal^20^. The crystal structure of the titania nanosheet^21^ consists of edge-shared TiO_6_ octahedra, which is similar to typical TiO_2_ crystals, e.g., anatase and rutile. The titanium atoms of the nanosheets are octahedrally-coordinated tetravalent, which was confirmed using electron energy-loss spectroscopy (EELS)^22^,^23^ as described in Supplementary Information. We successfully prepared free-standing titania nanosheets, and their crystal structure was revealed using conventional TEM observations^22^,^23^,^24^. Because the thickness of the titania nanosheet is only one titanium or two oxygen atoms, the attached dye molecules exhibited recognizable ADF contrast.

In this study, we directly observed the distribution of dye molecules on titania surfaces using low-voltage, low-temperature ADF imaging with quantitative intensity analysis. First, we investigated ADF images of a pristine titania nanosheet with atomic resolution. Then, we examined dye molecules on the titania nanosheets and their lateral distribution.

## Results and Discussion

**Figure 1:**
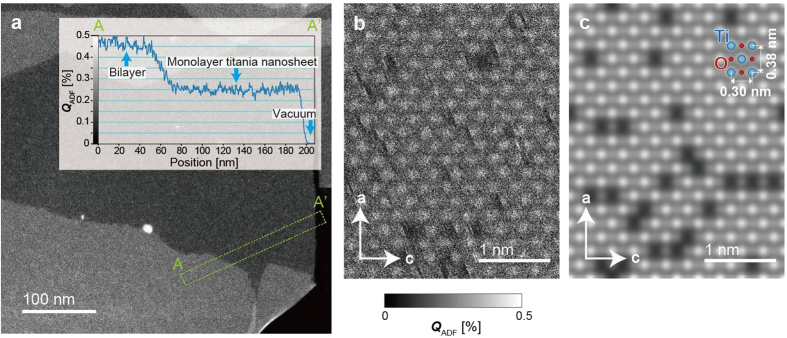
Experimental and simulated ADF images of titania nanosheet. (**a**) ADF image of monolayer titania nanosheet. The line profile of A–A′ is shown in inset. (**b,c**) Experimental (**b**) and simulated (**c**) ADF image of monolayer titania nanosheet. The directions of the a and b axes are indicated in (**b**,**c**). The intensity of these images is given as the quantitative ADF contrast *Q*_ADF_, and the *Q*_ADF_ scales for the experimental and simulated images were set in the same range of 0–0.5%. The mean quantitative contrasts of the monolayer titania nanosheet in (**a**–**c**) are 0.25 ± 0.01%, 0.21 ± 0.01%, and 0.28%, respectively.

Titania nanosheets in a colloidal suspension are surrounded by tetrabutylammonium (TBA, (C_4_H_9_)_4_N^+^) ions, which can be removed by photocatalytic reaction, resulting in free-standing nanosheets with clean surfaces (see Methods and Supplementary Figs S1–S4). Here, we confirm the crystal structure of the pristine titania nanosheets using high-resolution ADF imaging, which has not been previously achieved. Figure 1a presents the ADF images, whose quantitative contrasts *Q*_*ADF*_ were scaled by the ratio of the ADF signal to the incident electron intensity^18^. As demonstrated in the inset in Fig. 1a, the intensity profile along A–A′ exhibits a stepwise feature; the averaged intensities are 0%, 0.25%, and 0.46%, which are considered to be the vacuum, monolayer, and bilayer areas, respectively. The numbers of the titania nanosheet layers could be counted using quantitative ADF imaging.

Figure 1b,c present the experimental and simulated high-resolution ADF images, respectively. Because both ADF images are in the same contrast range (0–0.5%), we can directly compare these quantitative contrasts. The numerous bright dots in these ADF images correspond to Ti atoms, and Ti vacancies appear as dark areas. The averaged quantitative contrasts of the monolayer titania nanosheets are observed to be 0.21% and 0.28% of the experimental and simulated ADF images, respectively. Although the quantitative reproducibility of the ADF contrast in current simulation programs has not been fully established, the experimental averaged contrast is consistent with the simulated value. This finding indicates that clean surfaces of the titania nanosheets were prepared using our specimen preparation procedure, and the free-standing titania nanosheets could be used as substrates for dye molecules. The small differences in the averaged contrast values of a monolayer between Fig. 1a,b may be caused by a small amount of specimen contamination. If the attached materials originate from the carbon material, their thicknesses must be less than a monolayer because the averaged contrast of single-layer graphene was observed to be 0.05% in our previous studies^18^. The results of further investigation of the pristine titania nanosheets are provided in the Supplementary Information.

**Figure 2:**
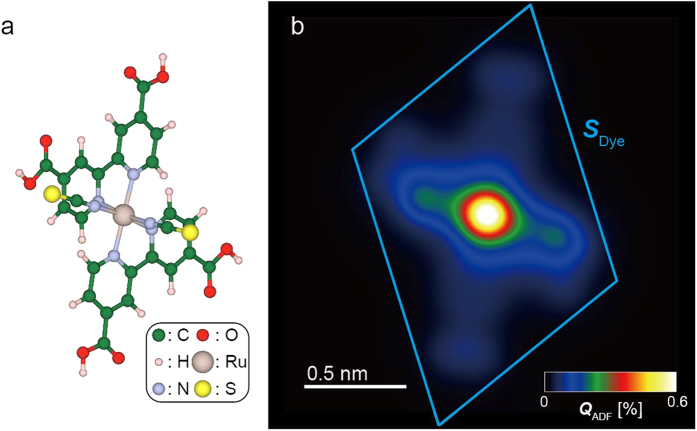
ADF image simulation of dye molecule. (**a,b**) Structural model^25^ (**a**) and simulated ADF image (**b**) of a dye molecule. The *Q*_ADF_ scale in (**b**) was set in the range of 0–0.6%. The parallelogram *S*_*Dye*_ in (**b**) corresponds to the area of a dye molecule on the surface.

To evaluate the detectability of the dye molecules, STEM image simulation of a single dye molecule (N3 dye, C_26_H_16_N_6_O_8_RuS_2_) was performed. A structure model of a dye molecule^25^,^27^, which is one of the possible binding configurations^8^ on a titania surface, and a simulated ADF image are presented in Fig. 2a,b, respectively. The area of the parallelogram *S*_*Dye*_ in Fig. 2b corresponds to the area of the dye molecule on a surface and equals 1.45 nm^2^; this value is necessary to estimate the coverage of dye molecules. The simulated image (Fig. 2b) shows the Ru atom position as a bright peak, whose quantitative contrast is 0.6% at the maximum. If dye molecules are attached on a titania nanosheet, the quantitative contrast proportionally increases with the coverage of the molecules because the incident probe is not greatly modified by the ultrathin specimen. Because the averaged quantitative contrast of the titania nanosheet was observed to be approximately 0.25% (Fig. 1), the intense Ru peak of the attached dye molecules can be recognized. Therefore, a single dye molecule is detectable in the ADF images when these molecules attached on titania nanosheets.

Here, we introduce the ADF scattering cross section of a single molecule to investigate its lateral dispersion. Table 1 lists the calculated ADF scattering cross sections of a Ru atom, other atoms (C, H, N, O, and S), and a whole dye molecule; the last value is a unit quantity of a single dye molecule *σ*_*Dye*_. As demonstrated in Fig. 2b, the maximum ADF contrast of the dye molecule is mainly attributed to the Ru atom because of its high atomic number, whereas the major part (68%) of the ADF cross section is attributed to the other atoms (carbon *et al.* see Table 1), which make up a high percentage of the molecular weight. For single-molecule coverage, which is equivalent to the two-dimensional close-packed arrangement, the averaged quantitative contrast *Q*_*Dye*_ is estimated to be 0.076% from (*σ*_*Dye*_/*S*_*Dye*_) × 100. The dye molecule coverage on titania nanosheets will be discussed using averaged quantitative contrasts in the following parts. We also calculated ADF scattering cross sections of other binding configurations, and they exhibited similar values (see Supplementary Fig. S6).

**Figure 3:**
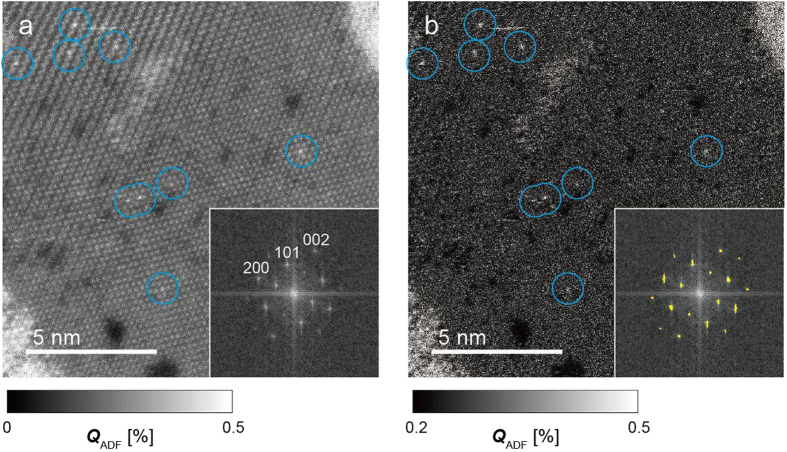
Dye molecule distribution on titania nanosheet. (**a,b**) Experimental ADF image (**a**) and Fourier filtered image (**b**) of dye molecules attached on a titania nanosheet. The insets show the Fourier transform patterns. The inset in (**b**) shows the masked areas to construct the Fourier filtered image. The *Q*_ADF_ scales of (**a**,**b**) were set to the ranges of 0–0.5% and 0.2–0.5%, respectively. Considering the monolayer titania nanosheet contrast, the ADF scattering cross sections of the open circular areas in (**b**) were estimated to be 0.0014 ± 0.0004 nm^2^. These values are consistent with the cross section of one N3 dye molecule (*σ*_*Dye*_ = 0.0011 nm^2^).

Figure 3a present a high-resolution ADF image of the dye molecules on a titania nanosheet. The presence of the dye molecules on titania nanosheets was also confirmed by EELS (see Supplementary Fig. S4). As indicated by the open circles, there are several bright peaks whose quantitative contrast is close to 0.8% at the maximum. The inset in Fig. 3a presents the Fourier transform of the ADF image, revealing the spots corresponding to the atomic lattice of the titania nanosheet. To clarify the dye-molecule contrast, all the spots were masked (see the yellow areas in the inset of Fig. 3b), and the inverse Fourier transformed image was calculated, as presented in Fig. 3b. The periodic contrasts of the titania nanosheet are reduced, and the bright peaks (open circles) are easily distinguished. Note that the averaged intensity is not changed by this image processing. We evaluated the ADF scattering cross section of each bright peak area, assuming an averaged intensity of the titania nanosheet of 0.21% (see Fig. 1b). The ADF scattering cross sections of the circular areas were found to be 0.0014 ± 0.0004 nm^2^. These values are comparable to the cross section of the dye molecule *σ*_*Dye*_ (see Table 1), namely, each circular area contains a single dye molecule. This analysis of the dye-molecule distribution becomes possible for the first time in this study. This procedure visualizes the distribution of dye molecules and, moreover, can be applied to distinguish the type of the attaching molecules.

**Figure 4:**
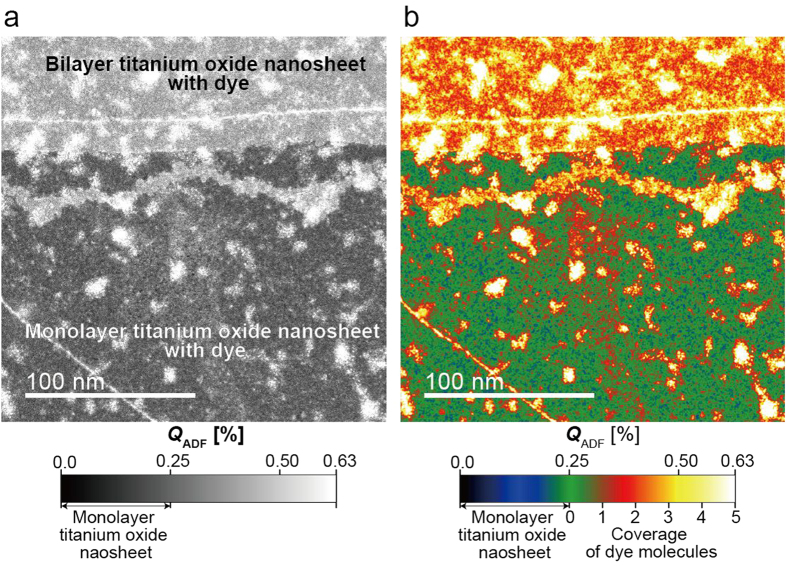
Dye molecule distribution map. (**a**) ADF image at low magnification. (**b**) Dye molecule distribution map. The *Q*_ADF_ scales in (**a**,**b**) were set in the same range of 0–0.63%. The coverage of N3 dye molecules is indicated using the ADF contrasts of the monolayer titania nanosheet (0.25%) and N3 dye molecule (0.076%).

It is also observed that the distribution of dye molecules is inhomogeneous; for instance, brighter domains are observed at the corners of Fig. 3. Next, we analyse the coverage of molecules including the bright domains. Figure 4a presents a low-magnification ADF image. The monolayer and bilayer titanium oxide nanosheets can be observed in the bottom and top regions, respectively. To clarify the coverage of attached dye molecules, the experimental ADF images were converted into a coloured image, assuming a monolayer titania nanosheet contrast of 0.25% (see Fig. 1a) and a single-molecule coverage contrast of 0.076%, as shown in Fig. 4b. To reduce shot noises, Fig. 4a was smoothed using a 7 × 7 pixel kernel, and the coverage of dye molecules can be recognized in Fig. 4b. The coverage of the majority of the surface was less than 1 molecule, and there were many aggregates with diameters of a few tens of nanometres with multi-molecule coverage. This microscopic observation of the dye-molecule distributions could accelerate the understanding of the attaching mechanism.

## Conclusion

We have investigated the lateral distribution of dye molecules for DSCs using low-voltage STEM, a quantitative ADF technique, a cooling specimen stage, and free-standing titania nanosheet substrates. Several technical difficulties such as a low signal, beam damage, and specimen preparation have been solved, and we have even visualized a single dye molecule. The quantitative contrast reveals the inhomogeneous dye-molecule distribution, in which the dye molecules aggregate and do not cover the majority of the surface at the early stage of the dye absorption. The present findings can help to optimize current DSC fabrication processes, and further improvements of the energy efficiency can be achieved by optimizing the dye-molecule distribution.

## Methods

### Specimen preparation

A titania nanosheet (Ti_0.87_O_2_) was derived by delamination of a K_0.8_Ti_1.73_Li_0.27_O_4_ layered titanate through a soft chemical procedure^20^,^26^. In this procedure, K^+^ and Li^+^ ions in the layered titanate were extracted, and negatively charged Ti_0.87_O_2_ layers were formed as colloidal sheets surrounded by tetrabutylammonium ions (TBA ions: (C_4_H_9_)_4_N^+^). A droplet of the aqueous solution containing the titania nanosheet/TBA ion was dropped on a holey carbon film of a TEM grid. Then, ultraviolet (UV) light was illuminated to decompose the TBA ions surrounding the nanosheets via a photocatalytic reaction. Consequently, a pristine titania nanosheet specimen on the TEM grid was prepared. A commercial N3 dye (*cis*-Bis(isothiocyanato)bis(2,2′-bipyridyl-4,4′-dicarboxylato)ruthenium(II), Sigma–Aldrich) was used as attaching molecules on the titania surface. Two TEM grids with titania nanosheets were prepared, and one was soaked in 0.01 mM dye molecules/ethanol solution to attach the molecules. The other was soaked in 99.5% ethanol solution. These two specimens, which were sufficiently dried, were used for STEM observations.

### ADF–STEM experiments and simulations

For the STEM observations, a Titan Cubed microscope (FEI) equipped with spherical aberration correctors (CEOS) was used at an acceleration voltage of 80 kV. Because titania nanosheets and dye molecules are more beam-sensitive than other nano-structured materials (e.g., graphene), low-voltage and low-temperature STEM observation was indispensable. A liquid-nitrogen cooling holder (UHRTR3500, Gatan) was used at −180 °C to reduce the contamination and irradiation damage of the specimens. The convergence semiangle of the incident probe was 20.7 mrad. The camera length for STEM imaging was set at 145 mm, and the inner and outer detection semiangles of the ADF detector were 48.4 and 200 mrad, respectively. The incident probe current, which was set to 10 pA, was measured using a bottom-mount charge-coupled device camera, whose conversion efficiency had been calibrated. The ADF images in this paper were acquired by a single exposure to prevent contaminations and irradiation damages. The obtained ADF image was converted into a quantitative contrast *Q*_ADF_ using our quantification procedure^18^.

The STEM image simulations were performed using a multislice program (xHREM with STEM Extension, HREM Research Inc.). The electron optical parameters, such as the convergence angle, defocus spread (20 nm), and ADF detector angles, were set to the experimental conditions. The effective source distribution of the incident probe size was assumed to be a Gaussian function whose full width at half maximum was 0.1 nm based on our previous results^19^ (see Supplementary Fig. S5).

## Additional Information

**How to cite this article**: Koshiya, S. *et al.* Microscopic observation of dye molecules for solar cells on a titania surface. *Sci. Rep.* **6**, 24616; doi: 10.1038/srep24616 (2016).

## Supplementary Material

Supplementary Information

## Figures and Tables

**Table 1:** Calculated ADF scattering cross sections of a Ru atom, other atoms (C, H, N, O, and S), and a dye molecule (C_26_H_16_N_6_O_8_RuS_2_).

	ADF scattering cross section [nm^2^]	percentage [%]
Ru	0.00035	32
C, H, N, O, S	0.00075	68
Dye molecule (C_26_H_16_N_6_O_8_RuS_2_)	0.00110	100
