# Supplementary material for: Microscopic observation of dye molecules for solar cells on a titania surface
Source: Sci Rep. 2016 Apr 18;6:24616. doi: 10.1038/srep24616 (PMC4834531; doi:10.1038/srep24616)
Supplement: Supplementary Information [file srep24616-s1.pdf]

Supplementary Information

Microscopic observation of dye molecules for solar cells on a titania surface

Shogo Koshiya [1,*], Shunsuke Yamashita [1,2] and Koji Kimoto [1,2,*]

[1] Surface Physics and Structure Unit, National Institute for Materials Science, 1-1 Namiki, Tsukuba, Ibaraki 305-0044, Japan.

[2] Department of Applied Chemistry, Kyushu University, 1-1 Namiki, Tsukuba, Ibaraki 305-0044, Japan.

Contents

1. Specimen preparation and preliminary analyses

Specimen preparation

Low-magnification scanning transmission electron microscopy (STEM) observation of pristine titania nanosheet

Dye-molecule distributions with varying soaking time

Elemental analyses of pristine and dye-molecule absorbed titania nanosheet

2. Details of annular dark-field (ADF) image simulation

3. Effect of various binding configurations on ADF image

References

* Correspondence and requests for materials should be addressed to S.K. and K.K. (emails: koshiya.shogo@nims.go.jp and kimoto.koji@nims.go.jp).

1. Specimen preparation and preliminary analyses

Specimen preparation

The scanning transmission electron microscopy (STEM) specimen preparation procedure is schematically illustrated in Fig. S1. After dropping the aqueous solution of titania nanosheets on a holey carbon film, illumination with ultraviolet (UV) light was performed for 2 h to photocatalytically decompose the tetrabutylammonium ions (TBA ions: $(C_4H_9)_4N^+$) on the nanosheets.[1] After soaking in the pure ethanol or dye molecule/ethanol solutions, the specimens were maintained in a vacuum desiccator under 1×10^3 Pa for complete drying. Because N3 dye molecules can be decomposed by visible light, the specimens were protected from intense light irradiation during the experiment.

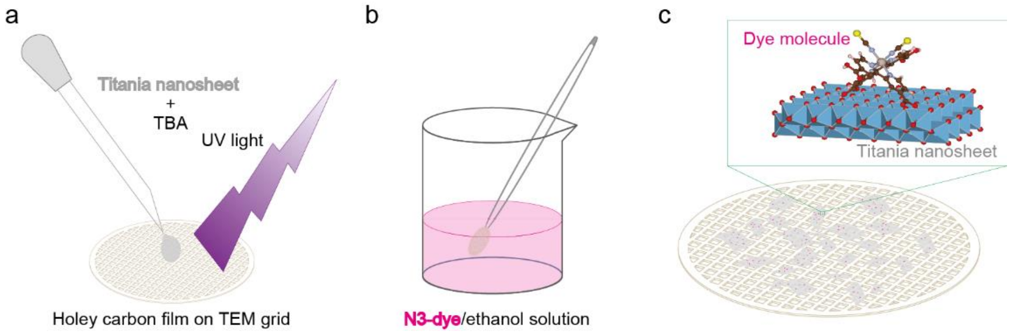

Figure S1. Schematic diagrams showing (**a**) the placement of the titania nanosheet onto a holey carbon film on a TEM grid, (**b**) the absorption of N3 dye molecules to the titania nanosheets, and (**c**) a specimen for STEM observations.

Low-magnification STEM observation of pristine titania nanosheet

Low-magnification STEM images of pristine nanosheets on a holey carbon film are presented in Fig. S2: (a) annular dark-field (ADF) and (b) bright-field (BF) images. The ADF image

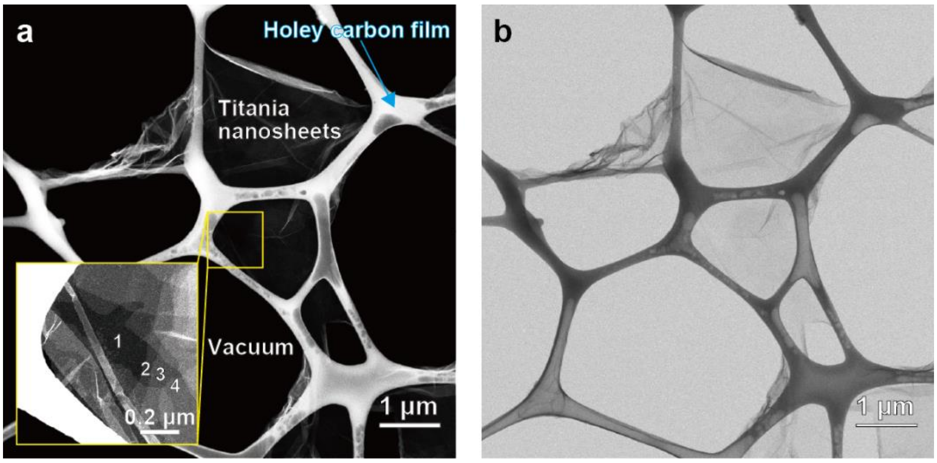

Figure S2. (**a**) ADF and (**b**) BF images of pristine titania nanosheet on holey carbon film in the same viewing field. The inset shows the magnified image with the number of titania nanosheets.

shows the reticular bright area and the less bright domains, corresponding to the holey carbon film and titania nanosheets, respectively. As observed in the inset of Fig. S2a, the number of titania nanosheet layers can be counted based on the ADF image contrast. However, the BF image does not show a clear contrast of the titania nanosheets because of the low scattering intensity of ultrathin specimens as well as of conventional TEM imaging. Thus, the titania nanosheet observation is quite challenging in TEM, and STEM-ADF imaging is more effective for the observation of titania nanosheets.

Dye-molecule distributions with varying soaking time

In the main text, we investigated an example of dye-molecule distribution, in which isolated and aggregated molecules were observed. It was the early stage of dye-molecule adsorption, and the amount of dye molecules could be controlled by changing the soaking time. Figure S3 presents ADF images of the specimens that were soaked in 0.01 mM dye molecule/ethanol solution for (a) 30 s, (b) 1 min, (c) 10 min, and (d) 100 min. The soaking time for Figs. 3 and 4 in the main text was 30 sec. Bright domains, which represent aggregated dye molecules, are observed even in the early stage of the absorption. We also observed that the edges of the titania nanosheets preferably absorbed dye molecules, which is similar to electrostatic absorption in a clay colloid system.[2] Although this phenomenon is not the major theme of this article, it provides a clue to accelerate dye-molecule adsorption for further investigation.

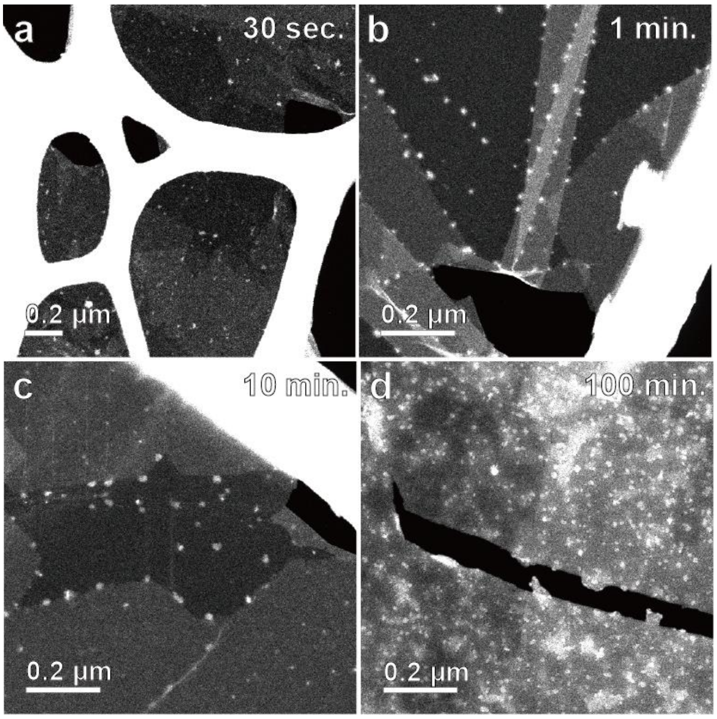

Figure S3. A series of ADF images showing dye molecule absorption on titania nanosheets with different soaking times ranging from 30 s to 100 min.

Elemental analyses of pristine and dye-molecule absorbed titania nanosheets

Electron energy-loss spectroscopy (EELS) was performed for elemental analysis using a post-column energy filter (GIF Quantum ERS, Gatan). The energy spread of the incident electrons was 0.9 eV for the full width at half maximum. Figures S4a and S4b present the EEL spectra of a pristine titania nanosheet, and Fig. S4c presents that of nanosheets with dye molecules. In Fig. S4a, Ti-*L* and O-*K* edges are observed; however, no N-*K* edge (at approximately 400 eV) is observed. These findings indicate that the TBA ions are fully decomposed, and the surface of titania nanosheets becomes clean. The titanium L_3 and L_2 edges indicate crystal-field splitting[3], as represented by the arrows in Fig. S4b, suggesting a tetravalent titanium atom surrounded by six oxygen atoms, i.e., a TiO_6 octahedron.[4] In Fig. S4c, the N-*K* edge is additionally observed, which originates from dye molecules attached on the titania nanosheets. Although these results were of relatively low energy resolution, high-resolution EELS analyses were reported elsewhere.[4,5,1]

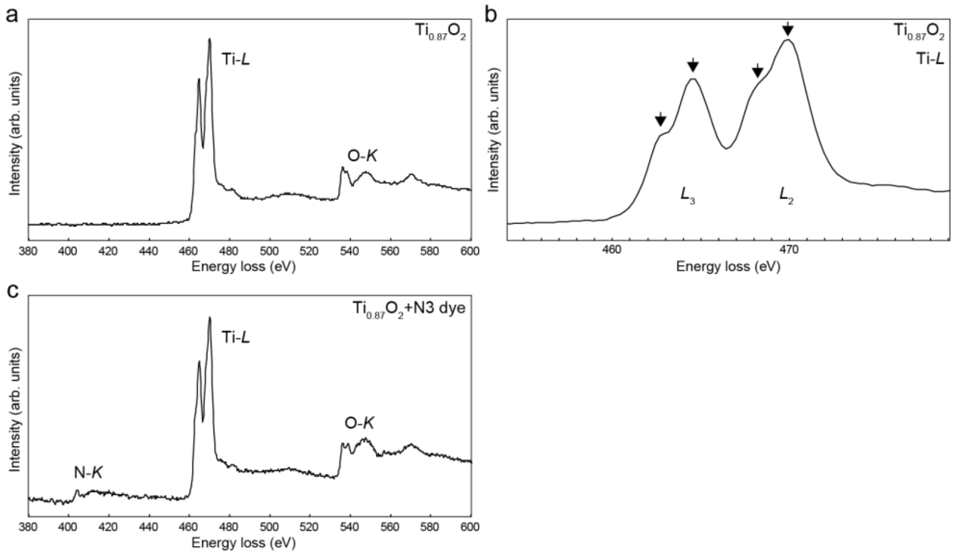

Figure S4. (**a**) and (**b**) EEL spectra of pristine titania nanosheet. (**c**) EEL spectrum of titania nanosheet with dye molecules.

2. Details of ADF image simulation

A multislice simulation program (xHREM with STEM Extension, HREM Research Inc.), which is based on the absorptive potential approximation for thermal diffuse scattering[6,7] was used. Figure S5a presents a simulated ADF image of a titania nanosheet under ideal conditions; the defocus spread of the objective lens and effective source distribution on the specimen were neglected. The bright dot, less bright dot, and dark area correspond to a titanium atom, double oxygen atoms, and a titanium vacancy, respectively. The averaged intensity of the ADF image is found to be 0.28%. To incorporate the effect of the defocus spread, 61 ADF images with varying defocus from -30 nm to +30 nm with 1-nm intervals were simulated, and a Gaussian-weighted averaged image was obtained. The effective source distribution, which is defined as the incoherent spread of the electron source, is incorporated via the convolution of the Gaussian function.[8] The detailed procedure for the image processing was provided in our previous paper.[8] Figure S5b presents such a processed ADF image incorporating the defocus spread and effective source distribution. In the processed ADF image (Fig. S5b), the position of oxygen atoms cannot be clearly observed. The experimental results can be directly compared with this simulated image, and the processed ADF image (Fig. S5b) well reproduces the experimental image (Fig. 1b). Note that the averaged intensity of the ADF images is not changed in either ADF image.

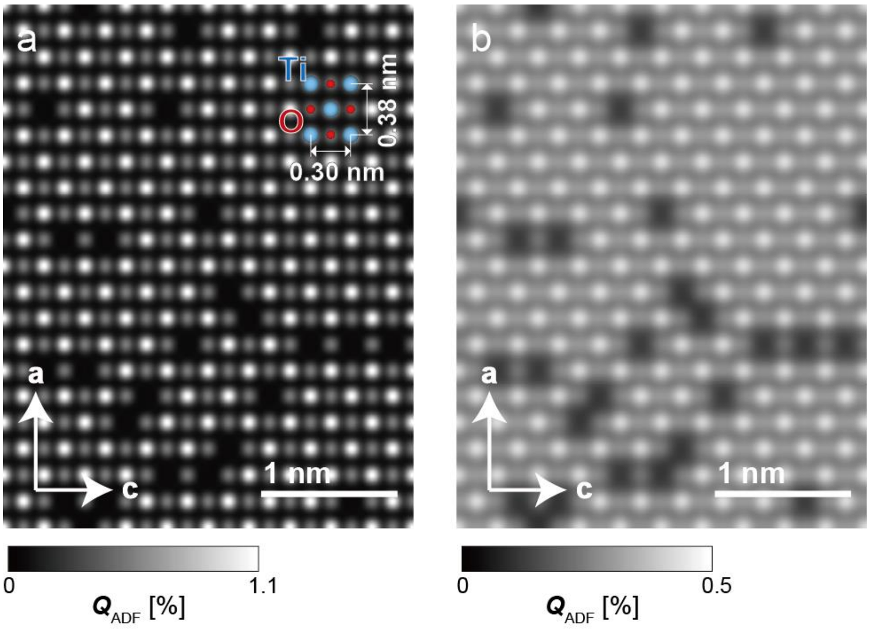

Figure S5. Simulated ADF images of a titania nanosheet (**a**) without and (**b**) with incorporating the defocus spread of the objective lens and the effective source distribution.

3. Effect of various binding configurations on ADF image

We assumed one binding configuration of a dye molecule on a titania nanosheet in the main text; however, other binding configurations have also been reported.[9,10] To evaluate the effect of the binding configurations, we elucidated their ADF scattering cross sections σ_{Dye} and averaged quantitative contrast Q_{Dye}. Figure S6 shows the schematic molecules and simulated ADF images of the different binding configurations. The ADF scattering cross sections σ_{Dye} are found to be almost independent of their binding configurations. The small differences in the ADF scattering cross sections are due to several overlapped atoms (see Figs. S6b and S6c), which can confine the incident electron, i.e., the atomic focuser.[11] The parallelograms S_{Dye} in the schematics correspond to projected areas. When dye molecules cover the entire titania nanosheet surface without overlapping, the averaged quantitative contrasts Q_{Dye} can be calculated as $(\sigma_{Dye}/S_{Dye}) \times 100$. The averaged quantitative contrasts for the different binding configurations agree within ±10%, as demonstrated in Fig. S6. Thus, the different binding configurations do not cause substantial differences in the averaged quantitative contrast and dye-molecule coverage.

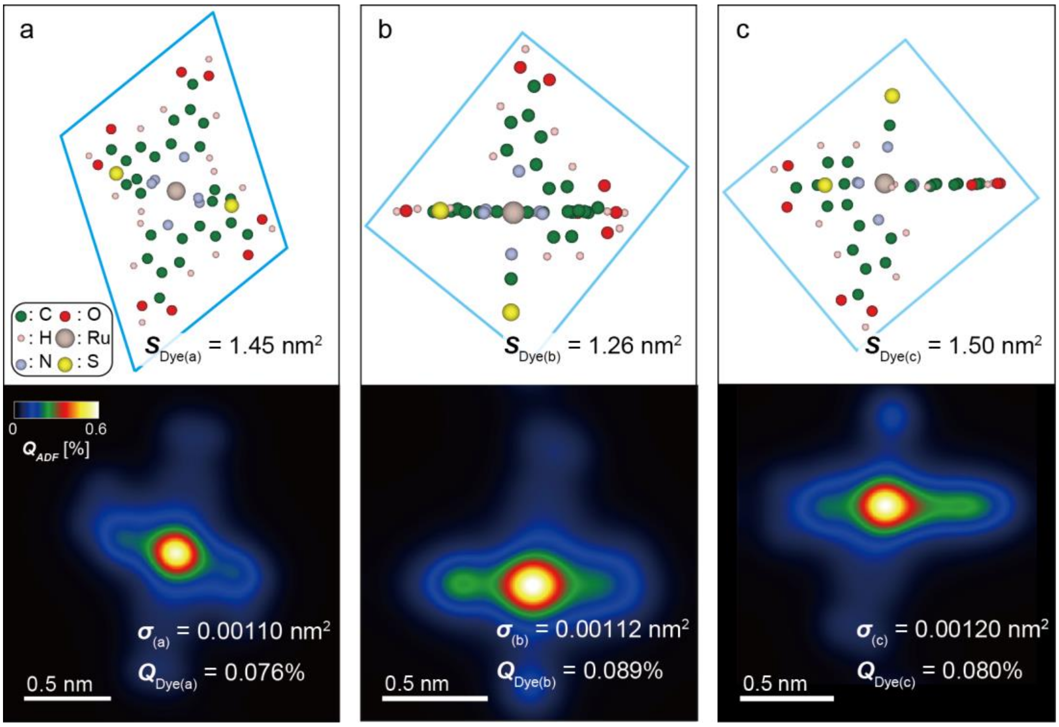

Figure S6. Structural models and simulated ADF images of dye molecules with different binding configurations. The area of the dye molecule S_{Dye}, ADF scattering cross section σ_{Dye}, and averaged quantitative contrast Q_{Dye} are indicated in each figure.

References

1. Ohwada, M., Kimoto, K., Mizoguchi, T., Ebina, Y. & Sasaki, T. Atomic structure of titania nanosheet with vacancies. *Sci. Rep.* **3,** 2801 (2013).
2. Olphen, H. van. *An introduction to clay colloid chemistry: For clay technologists, geologists, and soil scientists.* (Wiley, New York, 1977).
3. Egerton, R. F. *Electron Energy-Loss Spectroscopy in the Electron Microscope*. (Springer, New York, 2011).
4. Ohwada, M., Kimoto, K., Ebina, Y. & Sasaki, T. EELS study of Fe- or Co-doped titania nanosheets. *Microscopy* **64,** 77–85 (2014).
5. Ohwada, M. *et al.* Synthesis and atomic characterisation of a Ti_2O_3 nanosheet. *J. Phys. Chem. Lett.* **2,** 1820–1823 (2011).
6. Ishizuka, K. A practical approach for STEM image simulation based on the FFT multislice method. *Ultramicroscopy* **90,** 71–83 (2002).
7. Weickenmeier, A. & Kohl, H. Computation of absorptive form factors for high-energy electron diffraction. *Acta Crystallogr. Sect. A Observed. Crystallogr.* **47,** 590–597 (1991).
8. Yamashita, S. *et al.* Quantitative annular dark-field imaging of single-layer graphene—II: atomic-resolution image contrast. *Microscopy* **64,** 409–418 (2015).
9. Murakoshi, K. *et al.* Importance of binding states between photosensitizing molecules and the TiO_2 surface for efficiency in a dye-sensitized solar cell. *J. Electroanal. Chem.* **396,** 27–34 (1995).
10. Ikeda, M. *et al.* Lateral distribution of N3 dye molecules on TiO_2(1 1 0) surface. *J. Photochem. Photobiol. A Chem.* **202,** 185–190 (2009).
11. Cowley, J. M., Spence, J. C. H. & Smirnov, V. V. The enhancement of electron microscope resolution by use of atomic focusers. *Ultramicroscopy* **68,** 135–148 (1997).
